# LDDNet: A Deep Learning Framework for the Diagnosis of Infectious Lung Diseases

**DOI:** 10.3390/s23010480

**Published:** 2023-01-02

**Authors:** Prajoy Podder, Sanchita Rani Das, M. Rubaiyat Hossain Mondal, Subrato Bharati, Azra Maliha, Md Junayed Hasan, Farzin Piltan

**Affiliations:** 1 Institute of Information and Communication Technology, Bangladesh University of Engineering and Technology, Dhaka 1205, Bangladesh; 2 Faculty of Engineering and IT, The British University in Dubai, Dubai P.O. Box 345015, United Arab Emirates; 3 National Subsea Centre, Robert Gordon University, Aberdeen AB10 7AQ, UK; 4 Ulsan Industrial Artificial Intelligence (UIAI) Lab, Department of Electrical, Electronics and Computer Engineering, University of Ulsan, Ulsan 44610, Republic of Korea

**Keywords:** infectious disease, COVID-19, CT scan, X-ray, ResNet152V2, DenseNet201, XceptionNet

## Abstract

This paper proposes a new deep learning (DL) framework for the analysis of lung diseases, including COVID-19 and pneumonia, from chest CT scans and X-ray (CXR) images. This framework is termed optimized DenseNet201 for lung diseases (LDDNet). The proposed LDDNet was developed using additional layers of 2D global average pooling, dense and dropout layers, and batch normalization to the base DenseNet201 model. There are 1024 Relu-activated dense layers and 256 dense layers using the sigmoid activation method. The hyper-parameters of the model, including the learning rate, batch size, epochs, and dropout rate, were tuned for the model. Next, three datasets of lung diseases were formed from separate open-access sources. One was a CT scan dataset containing 1043 images. Two X-ray datasets comprising images of COVID-19-affected lungs, pneumonia-affected lungs, and healthy lungs exist, with one being an imbalanced dataset with 5935 images and the other being a balanced dataset with 5002 images. The performance of each model was analyzed using the Adam, Nadam, and SGD optimizers. The best results have been obtained for both the CT scan and CXR datasets using the Nadam optimizer. For the CT scan images, LDDNet showed a COVID-19-positive classification accuracy of 99.36%, a 100% precision recall of 98%, and an F1 score of 99%. For the X-ray dataset of 5935 images, LDDNet provides a 99.55% accuracy, 73% recall, 100% precision, and 85% F1 score using the Nadam optimizer in detecting COVID-19-affected patients. For the balanced X-ray dataset, LDDNet provides a 97.07% classification accuracy. For a given set of parameters, the performance results of LDDNet are better than the existing algorithms of ResNet152V2 and XceptionNet.

## 1. Introduction

Diseases that can be passed between humans or by animals or insects are communicable or transmissible diseases. The infectious organisms that cause these disorders include viruses, bacteria, fungi, etc. The most typical signs of such infections are fever and weakness, although these symptoms can vary depending on the organism that caused the infection. Most infections are not life-threatening; however, some are. The novel coronavirus disease, called COVID-19, is a life-risking transmittable disease and is caused by the severe acute respiratory syndrome coronavirus (SARS-CoV-2). In December 2019, it was discovered in Wuhan Province, China [1], for the first time. Since it is an individual-to-individual transmissible disease that spreads rapidly, it has created a pandemic. A healthy person can be infected by the coronavirus through droplets, or by inhaling aerosols containing the virus, or if the eye, mouth, or nose come into direct contact with an infected person’s cough, exhale, sneeze, or speech [2]. Therefore, to control the outbreak of the virus, it is highly recommended that if diagnosed in a person, he or she must be self-quarantined. Coughing, loss of smell, fever, absence of the sense of taste, and breathing complications are the most common signs of COVID-19. As the virus spreads from the infected person to those nearby [3,4,5], early detection of infected individuals is crucial so that they can isolate themselves and receive appropriate therapies for a speedy recovery.

Two types of testing kits are used to identify a COVID-19-infected person: antigen testing, which can detect a patient who is now sick, and antibody testing, which detects antibodies in the blood of a person who was previously infected with the coronavirus [3]. Most antigen tests use polymerase chain reaction (PCR) to identify COVID-19, and for this reason, the tests are called PCR tests. This RT-PCR test is carried out by RNA extraction from a swab collected from the back of the nose or throat as a clinical specimen [4]. However, the processes may take several hours; by this time, the virus may have spread to many uninfected people. Sophisticated lab equipment and technicians are also required for PCR tests. Moreover, the RT-PCR test is less sensitive for detecting COVID-19, which may result in many false negatives. Again, an incorrectly identified negative patient may contaminate a significant number of people by interacting with them. Hence, to minimize the risk of COVID-19, an improved diagnosis system is required that will result in fewer false negatives and can detect the presence of the coronavirus at the early stage of infection. 

To resolve this problem and accelerate the detection process, chest radiology imaging may be an alternative in the detection of COVID-19 [3], as respiratory symptoms are the first sign. Both chest computed tomography (CT) scans and chest X-rays (CXRs) show detailed images of soft tissues, bones, blood vessels, and internal organs of the chest, which is beneficial in detecting COVID-19 [6]. A COVID-19-infected person’s chest CT scan has some special characteristics, such as a peripheral distribution, fine reticular opacity, ground-glass opacities (GGOs), diffuse distributions, bilateral involvement, and vascular thickening [7]. During the first stage of screening, both CT and CXR have shown high sensitivity in detecting COVID-19 [8,9]. However, sometimes, radiologists’ visual tiredness may result in the failure to diagnose some minor lesions [10,11,12]. Considering this situation, artificial intelligence (AI)-based computerized diagnosis of COVID-19 is crucial. 

The rapid spread of COVID-19 and the critical necessity for early identification to minimize the incidence of COVID-19 among persons are the driving forces behind this investigation. Secondly, RT-PCR tests are limited in availability and require considerable time. Deep learning (DL), a subset of AI, plays a dynamic role in controlling the outbreak of the virus infection, not only by detecting the presence of the virus during the early stages but also by enhancing the public health care system and analyzing the virus for appropriate medications and vaccination [12,13,14,15,16,17,18,19,20,21,22,23,24,25,26]. To identify the presence of abnormalities in the lung, DL can be used for the reconstruction and segmentation of chest X-rays or CT scans [13,14]. Many research studies have provided accurate and effective results in the diagnosis of respiratory diseases using DL [15,16]. The requirement for more precise automated classification strategies for fast diagnosing COVID-19 patients is necessitated by CAD systems based on deep learning strategies. The literature review section shows that the effectiveness of DL algorithms depends on the datasets. Moreover, the availability of datasets limits the training of DL networks. Deploying optimization algorithms to select the optimal model architectures and hyper-parameters is also required. Thus, devising an algorithm that is suitable for both CT and X-ray images is important.

The main aim of this study is to enhance the classification of infectious diseases, such as coronavirus, pneumonia, etc. The main contribution of this paper is the development of a new deep learning (DL) framework termed optimized DenseNet201 for lung diseases (LDDNet) for data-driven diagnosis of COVID-19 and pneumonia lung diseases. To enhance the performance of this model, a combination of the global average pooling layer, batch normalization, dense layer, and dropout layer are attached to the base model. To overcome the overfitting and underfitting issues of deep neural networks, the early stopping method is created in LDDNet to halt the training of deep neural networks after a certain number of epochs in which the validation accuracy stops improving. This is particularly important as the process for selecting when to cease training deep neural networks can determine the generalization capability of a model; too many training epochs cause the model to overfit the training data, whereas too few training epochs result in underfitting. Then, the weights with the highest validation accuracy are restored and used for testing. Moreover, using open-access sources, three multiclass datasets of lung diseases are introduced in a new format, with one dataset containing 1043 CT images, one containing 5935 X-ray images, and the other being a balanced X-ray dataset of 5002 images. The proposed LDDNet model is applied to the datasets, the performance is compared with the optimized ResNet152V2 and optimized Xception models for a given set of parameters. Different optimizers, including Adam, Nadam, and SGD, are applied for proper diagnosis and evaluation of each class. Note that to enable a fair comparison with LDDNet, the optimized ResNet152V2 and optimized Xception models were also optimized using the same concept as LDDNet; however, for simplicity, the optimized ResNet152V2 is expressed as ResNet152V2, and the optimized Xception as Xception. 

The rest of the paper is organized as follows: Section 2 presents the relevant literature, and Section 3 describes the datasets. Next, Section 4 describes the architecture of the proposed LDDNet model. The results of the application of LDDNet to three different datasets are presented in Section 5. A comparison of the results of LDDNet with the existing models is also detailed in Section 5. Finally, Section 6 provides the concluding remarks and future research goals. 

## 2. Literature Review

Several research papers have reported the use of DL for infectious lung diseases. For example, a research article by Lawton et al. [10] evaluated the performance of transfer learning architecture on lung CT scans to identify the presence of coronavirus. In their study, the best results were observed by combining one of the transfer learning architectures, VGG-19, with the dataset using contrast-limited adaptive histogram equalization, with an accuracy of 95.75%. However, the use of histogram equalization was not an absolute technique because, in some cases, the architecture combined with the histogram equalization showed better performance, and sometimes the architecture without the histogram equalization showed higher accuracy. An AI-based study by A.A. Ardakani et al. [11] proposed a method for COVID-19 detection using a total of 1020 CT scan images of 108 COVID-19-affected patients and 86 non-COVID-19-affected patients (other viral phenomena diseases). In this study, Xception and ResNet-101 showed outstanding performance among 10 well-established conventional neural networks in detecting COVID-19 infection from the non-COVID-19 group, with a sensitivity of 98.04% and 100%, respectively. However, the dataset used in this work was very small. 

Ahuja et al. [3] introduced a deep transfer learning-based model for the detection of COVID-19 in lung CT images. To enhance the accuracy, they suggested a three-phase detection model. In the first phase, a stationary wavelet was used for data augmentation; in the next phase, a pretrained conventional neural network (CNN) was implemented for the detection of COVID-19; and in the final phase, the abnormality was localized in the CT slices. The authors claimed that the ResNet18 transfer learning-based classification model showed better accuracy, with 99.82% in the training, 97.32% in the validation, and 99.4% in the testing of the given datasets. The main limitation of this study is that the proposed model was not tested on a large CT scan dataset of coronavirus-infected patients. A stacked ensemble model was proposed by Jangam et al. [5]. In this study, the VGG-19 and DesNet-169 models were ensembled, and the authors claimed that this ensemble model showed better results than the other existing models for the case of five datasets, including three datasets of CT scan images and two datasets of X-ray images. Another study [17] proposed an ANN-based framework for the fast and automatic detection of patients infected by COVID-19. A study by Mukherjee et al. [18] claimed that if multiple data types were integrated, then more information could be found, which might be helpful in detecting the anomaly patterns of COVID-19. Their main objective was to observe whether a single deep neural network could train and test two different radiological image datasets. For this reason, they trained and tested CT scan images and X-ray images using a CNN-tailored DNN. The overall accuracy of the experiment was 96.28%. Another DL-based study was evaluated by Arora et al. [19] for the detection of COVID-19 from chest CT scans. In this work, a super-residual dense neural network was deployed to enhance the efficiency of benchmark datasets of lung CT scans, for example, SARS-CoV-2 and COVID-19. Compared to other models, the MobileNet model provided better results, with an accuracy of 94.12%.

A new DL-based algorithm was proposed by Bharati et al. [20] to analyze COVID-19 cases using X-ray images. The proposed algorithm, named CO-ResNet (optimized residual network), was developed by optimizing the conventional ResNet101, which was carried out by applying hyperparameter tuning. The proposed model was applied to a dataset of 5935 X-ray images and the result was better than the other existing conventional ResNet models. Another paper by Bharati et al. [21] introduced a modified neural architecture search network (NASNet) to diagnose coronavirus-affected patients from lung CT scans. NASNet-Mobile and NASNet-Large were implemented on a dataset containing 3411 lung CT scans, where 85% of the CT scan images were used for training the model and 15% of the images were used for testing. In this study, NASNet-Mobile showed an accuracy of 82.42% whereas NASNet-Large showed an accuracy of 81.06% for a similar number of epochs. From other literature as well, we receive several insights for the algorithmic improvements [26,27,28,29,30,31,32,33,34,35,36,37,38,39,40,41,42,43].

A self-developed architecture, CTNet-10, with an accuracy of 82.1%, was proposed by Shah et al. [35] to distinguish COVID-19- from non-COVID-19-affected patients in a CT scan dataset. Among some other tested DL architectures, e.g., VGG-19, VGG-16, DenseNet-169, ResNet-50, and InceptionV3, the highest accuracy achieved by VGG-19 was 94.52%. To detect the presence of COVID-19 from segmented 3D lung CT scan images, a weakly supervised DL-based software system was developed in [36]. Here, chest CT segmentation was carried out by a pretrained U-Net model. Then, the segmented 3D CT output was used as the input of a 3D DNN to predict the probability of the presence of COVID-19 in the CT scan. Then, the probability threshold was used to classify COVID-19-positive and -negative cases. Here, the used model achieved a 90.7% recall, 91.1% specificity, and 95.9% AUC. However, no temporal information was included in the lung segmentation, and inaccurate ground-truth masks were used for the training. Again, data were collected from a hospital in which no cross-center validations were used. Wang et al. [40] presented a 2D CNN model for the extraction of accurate features of COVID-19 and viral pneumonia from CT images. Nonetheless, extensive data integration and a low signal-to-noise ratio reduced the effectiveness of the employed model. The classification process was complicated due to the relatively high number of CT image parts that were unsuitable for the detection of pneumonia.

The authors of the research work [41] introduced a new DL-based method, CO-IRv2, which was derived from the InceptionNet and ResNetV2 methods, for the diagnosis of COVID-19. However, the authors only implemented this method for two-class classification. 

With their modified inception network, Wang et al. [42] improved the accuracy to an impressive 89.5% for CT images. Furthermore, they used independent samples to validate the excellent performance of the deep learning model they developed in this study, which achieved an accuracy of 79.3%. Furthermore, the suggested model acquired a sensitivity of 0.88 and 0.83 on the internal and external CT image datasets, respectively, making it a relatively effective screening tool. Moreover, the model showed improved performance for specific individuals, reaching an accuracy of up to 82.5%. Optimized inception ResNet V2 (IRV2) was proposed in [43] for the detection of COVID-19 on CT images. 

In [44], a novel Bayesian optimization-based convolutional neural network (CNN) model was proposed for the recognition of chest X-ray images, with a 96% success rate. There were two primary parts to the suggested paradigm. The first one learned and extracted deep features using a convolutional neural network. The second part was a Bayesian theory-based optimizer that finetuned the CNN hyperparameters based on some objective function [44]. In [45], the authors built three distinct DL models, including a suggested DL model with 13 fully connected dense layers, CNN, and an EfficientNetB7. In one scenario, COVID-19 was predicted using clinical data, while in the other scenario, CXR images were employed. In the third and last scenario, clinical data and CXR data were used for the prediction [45]. A framework consisting of Industry 4.0 techniques combining AI, cloud computing, and digital technologies was proposed in [46], and the CNN-based approach was improved for early detection and classification of patients into two classes (COVID-19 and normal), three classes (COVID-19, normal, and pneumonia), and four classes (normal, COVID-19, virus pneumonia, and bacterial pneumonia) [46]. In [47], four deep learning models (DenseNet121, ResNet50, VGG16, and VGG19) were trained to utilize the transfer learning approach to classify CXR images as either COVID-19 or normal. In the suggested study, both VGG16 and VGG19 performed better than the other two deep learning models [47]. The overall summary of the discussion is listed in Table 1. 

## 3. Description of the Dataset

**Figure 1:**
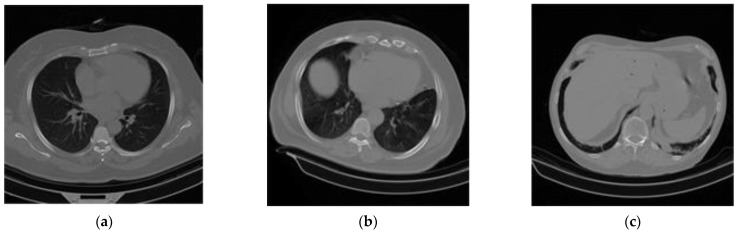
A sample of the experimental CT scan dataset: (**a**) normal or non-COVID-19, (**b**) COVID-19, and (**c**) community-acquired pneumonia (CAP).

This section describes the datasets considered for this research. To ensure comparable accuracy and rapidity in the screening and testing of COVID-19, the use of CT scans is satisfactory. In this paper, a large dataset of COVID-19, normal or non-COVID-19, and community-acquired pneumonia (CAP)-affected lung CT scan axial slices (along with their corresponding metadata) was built by curating data from [23,27,28,37,48,49,50,51,52]. As an example, curated images from each of the seven datasets are shown in Figure 1.

**Figure 2:**
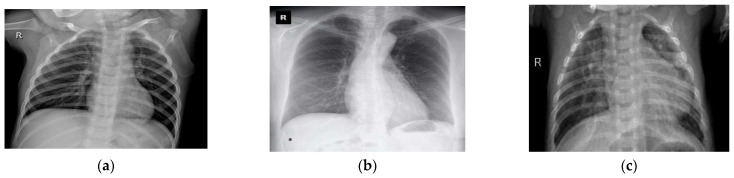
A sample of the experimental chest X-ray dataset: (**a**) normal or healthy chest, (**b**) COVID-19, and (**c**) pneumonia.

Chest X-ray images were also used to extend and enhance this experimentation. A total of 5935 X-ray images containing 4273 X-rays for pneumonia, 1583 X-rays for healthy chests, and 79 X-rays for COVID-19-affected chests were collected from two separate open-access sources [33,34]. Curated X-ray images from both datasets are shown in Figure 2. After this, 85% of the datasets were used for training and 15% for testing. In DL applications, i.e., in the COVID-19 diagnosis literature, these datasets have been publicly used, and their efficiency and effectiveness have also been proven. In the case of training, if these merged datasets can be used, then the generalization of various DL models is expected to improve. The details of the dataset are listed in Table 2 and Table 3. After that, a balanced X-ray dataset is taken into consideration [51]. This balanced dataset is actually a collection of four different open-source datasets of X-ray images [53,54,55,56]. This dataset has undergone some preliminary processing, such as the merging of the datasets and the resizing of the images. In the end, we shuffled the data and converted all of the images from grayscale to RGB format.

## 4. Proposed LDDNet Framework

This experiment was carried out in several stages. Normalization and data resizing were performed to avoid overfitting and simplify the generalization. Then, the dataset was separated into two portions, namely the training portion and the testing portion. We trained LDDNet and the existing ResNet152V2 and XceptionNet models with training data. For the experiment, data were collected for up to 30 epochs. Within 30 epochs, our proposed model achieved the best accuracy. The batch size was set to 32 when using the CT scan dataset for our experiment. However, the batch size was set to 64 for the X-ray dataset because the number of X-ray images in the dataset was large. It can be noted that appropriate batch sizes in conjunction with a suitable optimizer and hidden layers will unquestionably yield the highest results. There is no ideal batch size, so we must test and experiment with a variety of batch sizes. In addition to batch size, we must also consider the different optimizers to test. For instance, if we treat all samples as a single batch, we must perform a great deal of computation (which will be time-consuming and costly). Moreover, if we go with a larger batch size (with a suitable optimizer and hidden layers), it will unquestionably yield the highest results. The batch size has been adjusted to 32 for our experiment utilizing the CT scan dataset. In contrast, the batch size for the X-ray dataset has been set at 64, since the number of X-ray pictures in the dataset is substantial. This batch size has produced good results compared to the batch sizes of 16 or 32. Afterward, finetuning of the hyperparameters was performed on the model. To accelerate the overall performance of LDDNet, the pooling layer, batch normalization, dense layer, and dropout layer were applied after the base DenseNet201 model. For example, dense layers can avoid underfitting, whereas overfitting is avoided using data augmentation and dropout, which are described later. Batch normalization was used to accelerate model training. Then, the overall system was observed with regard to the accuracy, recall, precision, confusion matrix, F1 score, AUC, and receiver operator characteristic (ROC) curve values. 

### 4.1. Training Details

The training contexts and some parameters that were constant throughout this work are as follows:

- Framework: Tensorflow.
- Number of epochs: 30.
- Learning rate: 0.002.
- Loss function: cross-entropy loss.
- Batch size: 32 (for CT images) and 64 (for X-ray images).
- Optimizer: Adam, Nadam, SGD.

**Figure 3:**
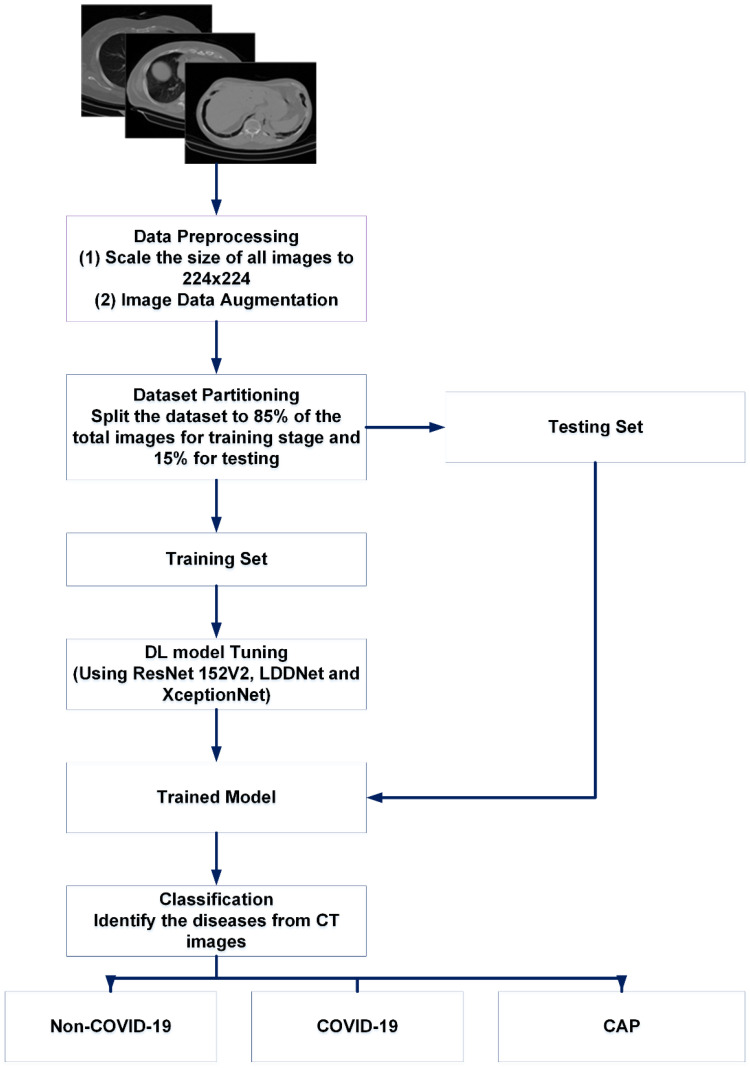
Basic summary of our proposed work for CT scan images.

**Figure 4:**
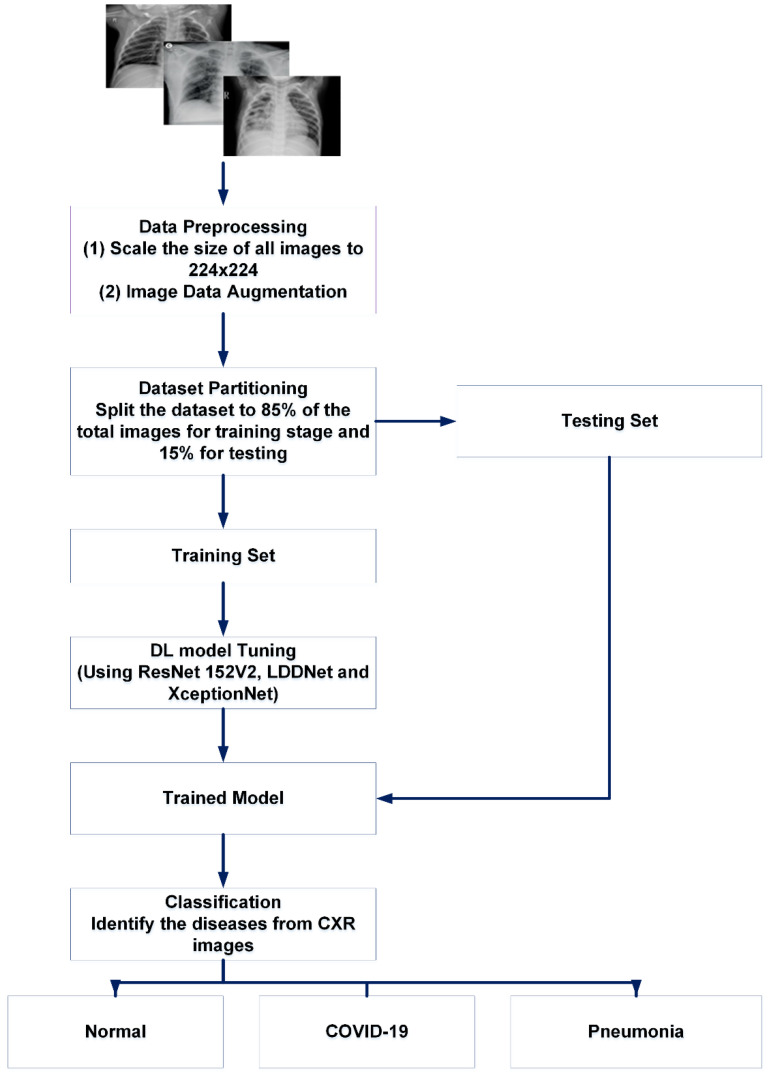
Basic summary of our proposed work for X-ray images.

The main procedure of our proposed system is depicted in Figure 3 and Figure 4.

### 4.2. Data Preprocessing

To optimize the DL models, the hyperparameters were tuned over multiple steps, including normalization and data augmentation. To fix the numerical columns of a dataset using a common scale, data normalization is crucial. Using normalization, model training can be accelerated, and the possibility of a stable gradient descent is also increased. The sample CT scan and X-ray images were different sizes. Therefore, the images were resized to 224 × 224 pixels using the RGB color. Pixel value normalization was completed between 0 and 1. The grayscale photos in the dataset were rescaled by multiplying the pixel values by 1/255. To increase the sample size for better training, data augmentation was applied to widen the data range. Various data augmentation strategies were used on the training set using the image data generator function of the Keras library in Python to prevent overfitting and boost the diversity of the dataset. Scale transformation was used to utilize lower pixel values within the same range, hence reducing the computational cost. With the help of the parameter value (1/255), each pixel’s value ranged between 0 and 1. Therefore, an angle of 15 degrees was utilized to rotate the images using the rotation transformation. The zoom range argument was used to accomplish the random zoom transformation: a value larger than 1.0 indicates that the images were enlarged, and a value less than 1.0 indicates that the images were shrunk. Therefore, a zoom range of 0.2 was utilized to enlarge the image. Flip was used to flip the image horizontally and vertically. The tuned hyperparameters of the model included the learning rate (LR), batch size (BS), epochs, optimizer, and dropout rate (DR). The following parameters were adjusted to get the best possible results from the experiment: batch size, epochs, DR, and initial LR were all set to 32 for the CT dataset and 64 for the X-ray dataset, 30, 0.5, and 0.002, respectively.

### 4.3. Model Architecture

This section describes the proposed LDDNet framework. For clarity, the existing models, such as ResNet and XceptionNet, are described before the description of LDDNet. Residual network, briefly known as ResNet, is an artificial neural network, where the network is formed by stacking residual blocks one after another at the top of the network. Xception stands for an extreme version of inception because it accepts the concept of inception to the extreme. In the inception model, to compress the original input, 1 × 1 convolutions are used first. Then, several types of filters are used to determine the depth of each input space. 

The proposed LDDNet uses a base model called DenseNet201, which solves the vanishing gradient problem, and traditional DenseNet was developed to improve the deterioration in the accuracy. The vanishing gradient problem is solved in DenseNet201 by modifying the architecture of standard CNN and simplifying the connections among layers. DenseNet is a convolutional network that helps the DL network go deeper but ensures that the connection between the layers is shorter to make the network more efficient for training. The layers of the DenseNet network are connected to all other deeper layers of the network. This means that the first layer is connected to all subsequent deeper layers of the network, the second layer is connected to all subsequent deeper layers, etc. This means that for a DenseNet architecture with N layers, there is a total of N(N + 1)/2 direct connections. In this process, the maximum amount of information can be transferred from the input layer to the output layer. All layers take inputs from their preceding layers and provide their feature maps to all succeeding layers to preserve the feed-forward nature of the network. DenseNet concatenates the outgoing feature maps with the incoming feature maps of the layer rather than the sum, which is unlike ResNet. However, it also has the same problem, such as ResNet, in that the dimensions of the concatenated feature maps are different. Hence, DenseNet is divided into DenseBlocks, where the filter numbers may vary within a block, but the dimensions of the feature maps are constant. Batch normalization is applied to the transition layers, which are the layers among the blocks, to reduce the current number of channels by 50%.

**Figure 5:**
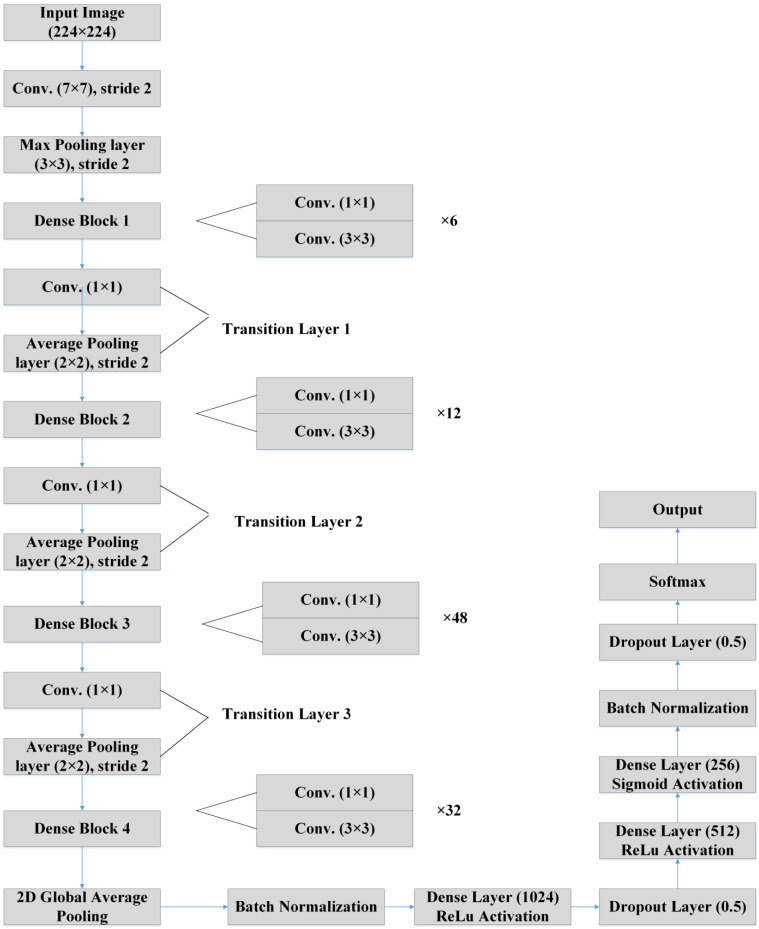
LDDNet architecture [57].

However, DenseNet needs fewer parameters, and it permits feature reusing, making the networks more compact. Again, DenseNet has achieved better results for competitive datasets and has shown state-of-the-art performances. Figure 5 depicts the basic building blocks of our LDDNet architecture, where global average pooling, batch normalization, a dropout layer, a dense layer, etc., are applied after the base model to enhance the model’s performance. Furthermore, Table 4 presents the dimensions of different layers of LDDNet.

To ensure that our proposed LDDNet is robust, we used several components, including batch normalization, a convolutional layer, a pooling layer, an activation function, a dense layer, a dropout layer, etc., as discussed below.

#### 4.3.1. Batch Normalization Layer

The batch normalization technique makes deep neural networks faster and more stable by normalizing the layer inputs. During the training of a deep network, it stabilizes the learning process and dramatically reduces the number of epochs required to train the network.

#### 4.3.2. Pooling Layer

The pooling layer aims to reduce the computation costs by decreasing the dimension of feature maps convolved from the convolutional layers. This layer minimizes the number of parameters while training the network. There are several types of pooling operations depending on the method used: max pooling provides the maximum value from the input elements, average pooling provides the mean value from the input elements, and sum pooling provides the summation of the input elements. 

#### 4.3.3. Activation Function

The activation function is used to approximate and learn all types of complex and continuous relationships among the variables of the convolutional neural network. It also decides which information in the model needs to be transferred in the forward direction and which information does not. It incorporates nonlinearity into the network. Some of the commonly used activation functions are ReLu, sigmoid, softmax, etc. Compared to other activation functions, the computational costs of ReLu are low, and the gradient convergence is also good. For the negative input, ReLu provides zero output and for the positive input, the output is the same as the input [52,57,58]. The mathematical equation for ReLu is:

(1) $$\mathrm{ReLu}\left(w\right)=\{\begin{array}{c} w,\;\;w>0\\ 0,\;\;w\leq 0 \end{array}$$

#### 4.3.4. Dense Layer

The dense layer is connected densely, meaning that the neurons of a layer are coupled with every neuron of the previous layer. A dense layer feeds all the outputs from its preceding layer and provides outputs to the next layer. 

#### 4.3.5. Dropout Layer

If all features are connected to the fully connected layer, this may result in overfitting for the training dataset. To overcome this problem, some neurons are dropped from the network while training the model, and this is carried out using the dropout layer. A dropout of 0.3 means that in the neural network, 30% of the neurons are dropped randomly. 

#### 4.3.6. SoftMax

In a neural network for multiclass classification, softmax is the name of the final output layer. SoftMax normalizes the network’s output to between one and zero. The softmax function calculates the probability for each class. The softmax activation function is calculated using this function:

(2) $$\mathrm{softmax}\left(x_{i}\right)=\frac{e^{x_{i}}}{\sum_{j}^{\;}\;e^{x_{j}}}$$

where x is the neuron values of the output layer and the exponential is used for nonlinearity. Normalization is carried out by dividing the exponential values by the sum of the exponential values. Finally, these values are converted into probabilities. 

In the proposed LDDNet, the features of the last deep layer can be projected by all the previous set of layers (this means that the deep network layers can reuse all the features produced by the previous layers). Typically, the size of the feature maps is down-sampled by half after the convolution layers in traditional CNNs’ architectures. Therefore, a variety of sizes may result from the aggregation of feature maps around the down-sampling layers. To address this problem, dense blocks were conceived of ahead of the down-sampling layers, with the dense block layers tightly interconnected; this keeps the size of the feature maps uniform across all dense blocks and reduces their size by a factor of two after down-sampling. In contrast to a regular convolutional network, which has Y nodes, the total number of linkages between the nodes in a dense block is Y(Y + 1)/2. However, if the layers are deep, the computation will be massive due to the large number of combined feature maps entered in the layers. Each 3 × 3 convolution layer was preceded by a 1 × 1 bottleneck layer to reduce computational overhead, and transition layers were included to greatly enhance network compactness by regulating the number of output feature maps. It can be noted that if the output size of the first convolution layer is 112 × 112, the size of the next max pooling layer will be 56 × 56. The output size of four dense blocks will be 56 × 56, 28 × 28, and 14 × 14, 7 × 7, respectively.

### 4.4. Optimizer

Optimizers are methods or algorithms that are used to minimize model losses by varying the learning rate, weight, or other types of attributes. Several types of optimizers were used in our experiments. Adaptive moment estimation (Adam) works with first-order and second-order momentums [39]. Nadam (Nesterov-accelerated adaptive moment estimation) is a combination of NAG and Adam optimizers, which was developed for a noisy gradient. The exponential decay of moving averages for current and previous gradients is added to accelerate the learning rate (α). The stochastic gradient descent randomly selects data from the dataset instead of taking all data at each iteration to reach the local minima. The SGD optimizer updates the weight.

The input image is processed in LDDNet by first passing through the DenseNet201 layers, which comprise the batch normalization and Relu layers, and then moving on to the transition layer. After the output is processed, 2D global average pooling is carried out, and then batch normalization is performed. After, there are a total of 1024 dense layers with Relu activation. Following this, there is a dropout layer of 0.5. Once more, the output is transmitted to 512 dense layers using the Relu activation method, and then it is sent to 256 dense layers using the sigmoid activation method. After this, the batch normalization step, the dropout layer with a rate of 0.5, and, finally, the Softmax layer are performed.

### 4.5. Pseudocode

To further clarify the proposed LDDNet framework, the following pseudocode of LDDNet is presented below.
*1**Collect the dataset and define its path, epoch, and batch size**2**Extract images and class labels from existing files**3**Normalize the pixel value array**4**During network initialization, randomly determine a startup weight.**5**Choose the initial pair of exercises**6**The forward calculation, comprising the subsequent steps:*
 *a.**Connect the input to the network.*
 *b.**Figure out the result for each neuron from the input layer to the output layer through the hidden layer.*
 *c.**Figure out the error at the output.**7**Countdown with the subsequent steps*
 *a.**Use the result error to calculate the error signal for the pre-output layer.*
 *b.**Use the error signal to calculate the weight correction.*
 *c.**Make appropriate adjustments to weight*
 *d.**Repeat the calculation in reverse for the remaining exercise pairs*
 *e.**Perform periodic network performance evaluations**8**Iterate the calculation until the network reaches the desired exit.*

## 5. Result and Analysis

In this section, the experimental results of the CT scan images are described, and then the results of the X-ray images are described. All experiments, including data preprocessing and analysis, were performed on the Google Cloud computing service Google Colab (colab.research.google.com (accessed on 25 December 2022)) using the programming language Python (version 3.7.15) and the framework Tensor Flow. In this experiment, three types of optimizers (Adam, Nadam, and SGD) were used with the same learning rate of 0.002. Results were obtained for LDDNet, and two existing models named ResNet152V2 and XceptionNet, in which all types of optimizers were implemented for each model. All results were calculated for 30 epochs. However, the batch size differed, with 32 for the CT images and 64 for the X-ray images, as the number of X-ray images was more than 5000. The results of the experiment were analyzed using some performance metrics, i.e., recall, F1 score, accuracy, PPV, confusion matrix, and ROC curve. 

If the lung of a suspected patient is not affected by coronavirus, then the result is negative, and the result is positive if the lung is infected by coronavirus. The results of this test of all COVID-19-affected persons may or may not be the same as the actual report of the infected persons. In the confusion matrix, four types of elements are used. True positive (TP) stands for correctly identified COVID-19-affected patients. False positive (FP) denotes non-COVID-19 patients mistakenly detected as COVID-19 positive. True negative (TN) indicates COVID-19-negative patients who are correctly detected as non-COVID-19. The false negative (FN) term represents non-infected patients who are incorrectly identified as COVID-19 positive. 

Accuracy is the performance measurement of correctly classifying a usual case as usual and an unusual case as unusual. The proportion of correctly identified COVID-19-positive cases to the sum of predicted COVID-19-positive cases is called the recall. The number of properly classified positive cases divided by the total number of predicted positive cases is called the precision. It is also called the positive predictive value (PPV). The harmonic mean of the precision and recall is called the F1 score. The area under the curve of the receiver operator characteristic (AUC-ROC) curve shows the performance of the classification model for all classification thresholds by plotting the true positive rate (TPR) in contrast to the false positive rate (FPR). A higher AUC-ROC value implies better performance of the model in classifying the true positive patients as positive and true negative patients as negative. 

### 5.1. Results and Analysis for the CT Scan Images

The results were analyzed for some infectious diseases affecting the lung, i.e., COVID-19, community-acquired pneumonia (CAP), and healthy or non-COVID-19. Table 5 shows the comparison among the three models for the Adam optimizer. All models classified the CAP phenomena accurately. However, in the case of COVID-19 and non-COVID-19, ResNet152V2 and LDDNet were classified more accurately, that is, 99.36% for both models, but this value was comparatively lower for XceptionNet, which was 96.18%.

Table 6 shows the comparison for the Nadam optimizer, where the accuracy of ResNet152V2, LDDNet, and XceptionNet in detecting COVID-19 is 97.45%, 99.36%, and 96.82%, respectively. For Nadam, LDDNet shows a higher accuracy of 99.36%, whereas ResNet152V2 and XceptionNet provide a 97.45% and 96.82% accuracy, respectively.

Considering the SGD optimizer, LDDNet shows a better accuracy rate of 98.73% for both COVID-19 and non-COVID-19 cases and 100% for CAP. The overall accuracy for ResNet152V2, LDDNet, and XceptionNet was 94.90%, 98.73%, and 93.63%, respectively, as shown in Table 7.

**Figure 6:**
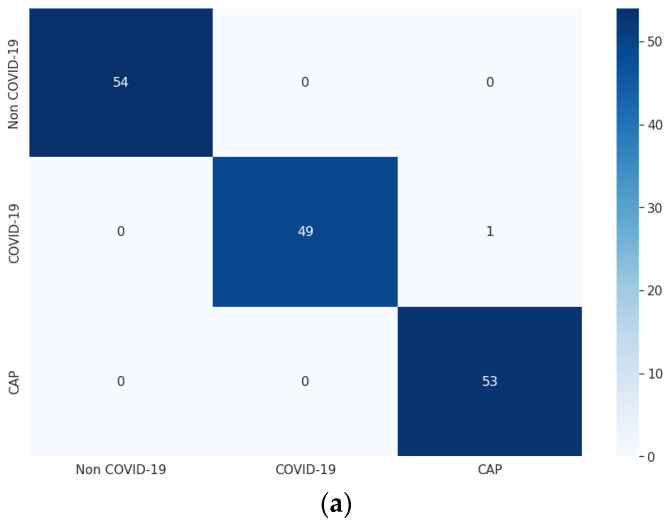

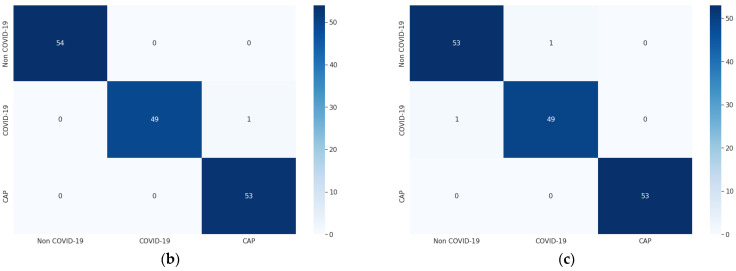
Confusion matrix for CT images using LDDNet: (**a**) Adam optimizer, (**b**) Nadam optimizer, and (**c**) SGD optimizer.

For each of the three optimizers, the overall model accuracy of LDDNet is higher compared to ResNet152V2 and XceptionNet. The confusion matrix of LDDNet for the Adam, Nadam, and SGD optimizers is represented in Figure 6, indicating the following:

- The Adam optimizer correctly classified all non-COVID-19 and CAP samples, though one COVID-19 sample was misclassified as non-COVID-19.
- The Nadam optimizer also correctly classified all non-COVID-19 and CAP samples; however, one COVID-19 sample was incorrectly classified as CAP.
- SGD inappropriately classified one non-COVID-19 CT scan as COVID-19 and one COVID-19 as non-COVID-19; however, it correctly classified all CAP CT scans.

From the confusion matrix, the model accuracy was 99.36%, 99.36%, and 98.72%, and the model error was 0.63%, 0.63%, and 1.27% for the Adam, Nadam, and SGD optimizers, respectively.

For the XceptionNet model, the model accuracy is 96.17%, 96.81%, and 93.63%, and the model error is 3.82%, 3.18%, and 6.36% for the Adam, Nadam, and SGD optimizers, respectively.

**Figure 7:**
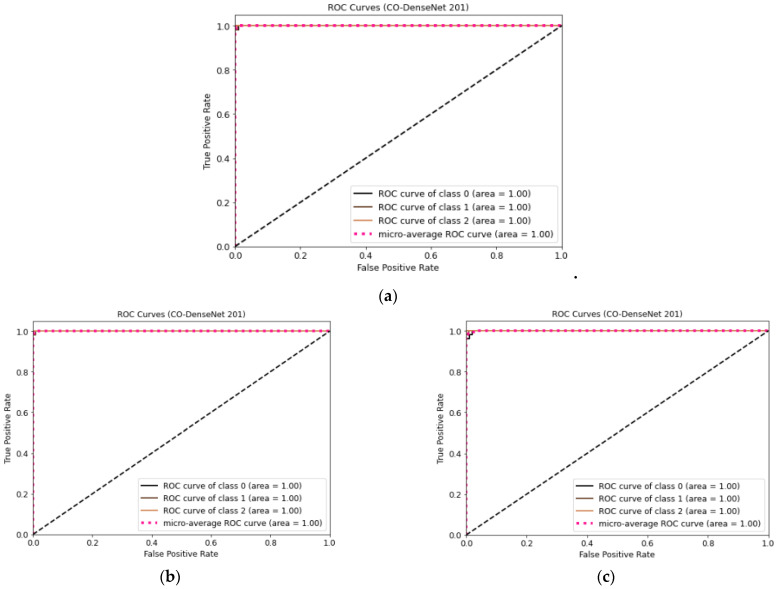
(**a**) ROC curve for CT images using LDDNet with Adam optimizer, (**b**) ROC curve for CT images using LDDNet with Nadam optimizer, and (**c**) ROC curve for CT images using LDDNet with SGD optimizer.

Figure 7 shows the ROC curve for LDDNet for the cases of the Adam, Nadam, and SGD optimizers. Here, class “0” indicates non-COVID-19, “1” indicates COVID-19, and “2” indicates CAP-affected CT scan images. From Figure 7, it is observed that the ROC values for all types of lunges are 100%, regardless of the optimizer used. 

### 5.2. Results and Analysis for X-ray Images

Three types of lung X-ray images, including normal or healthy lung, pneumonia-affected lung, and COVID-19-affected lung, are taken into consideration for the experimental analysis. Table 8 shows the comparison among the three models for the Adam optimizer. Here, the XceptionNet model shows the highest accuracy in the case of detecting COVID-19, which is 99.78%, whereas the accuracy for ResNet152V2 and LLDL is 99.55% and 99.21%, respectively. Even the PPV, recall, and F1 score of XceptionNet in COVID-19 detection are higher compared to the other implemented models. The model accuracy for ResNet152V2, LDDNet, and XceptionNet is 95.74%, 96.41%, and 94.5%, respectively. 

Table 9 shows the comparison for the Nadam optimizer, where the XceptionNet model provides the highest accuracy in detecting COVID-19. The accuracy of ResNet152V2, LDDNet, and XceptionNet is 99.21%, 99.55%, and 99.66%, respectively. However, the overall model accuracy for ResNet152V2, LDDNet, and XceptionNet is 95.17%, 96.52%, and 94.39%, respectively.

The results of the different performance matrices for the SGD optimizer are shown in Table 10, where LDDNet shows the highest accuracy in diagnosing COVID-19, with 99.44% and 98.32% for ResNet152V2 and 98.32% for XceptionNet. Here, the overall model accuracy for ResNet152V2, LDDNet, and XceptionNet is 91.81%, 95.06%, and 88.78%, respectively.

In the case of X-ray images, for each of the three optimizers, compared to ResNet152V2 and XceptionNet, the LDDNet model always shows the highest overall model accuracy. 

**Figure 8:**
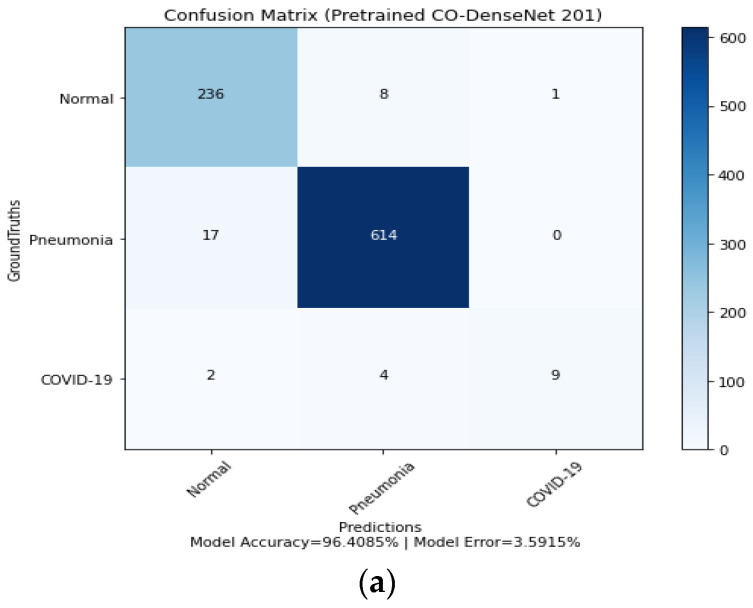

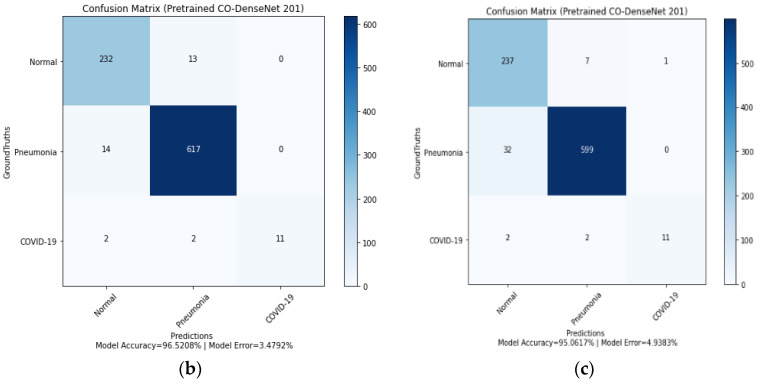
Confusion matrix for LDDNet: (**a**) Adam optimizer, (**b**) Nadam optimizer, and (**c**) SGD optimizer.

For the ResNet152V2 model, the model accuracy is 95.73%, 95.17%, and 91.8%, and the model error is 4.26%, 4.82%, and 8.19% for the Adam, Nadam, and SGD optimizers, respectively. The confusion matrix of LDDNet shown in Figure 8 indicates the following:

- The Adam optimizer can correctly classify 236 normal, 614 pneumonia-affected, and 9 COVID-19-affected lung X-ray images.
- In the case of the Nadam optimizer, 232 normal, 617 pneumonia-affected, and 11 COVID-19-affected lung X-rays are correctly classified.
- In implementing the SGD optimizer, 11 COVID-19-affected patients, 599 pneumonia-affected patients, and 237 normal lung X-ray images were appropriately classified. From the confusion matrix, the model accuracy is 96.40%, 96.52%, and 95.06%, and the model error is 3.59%, 3.47%, and 4.93% for the Adam, Nadam, and SGD optimizers, respectively.

**Figure 9:**
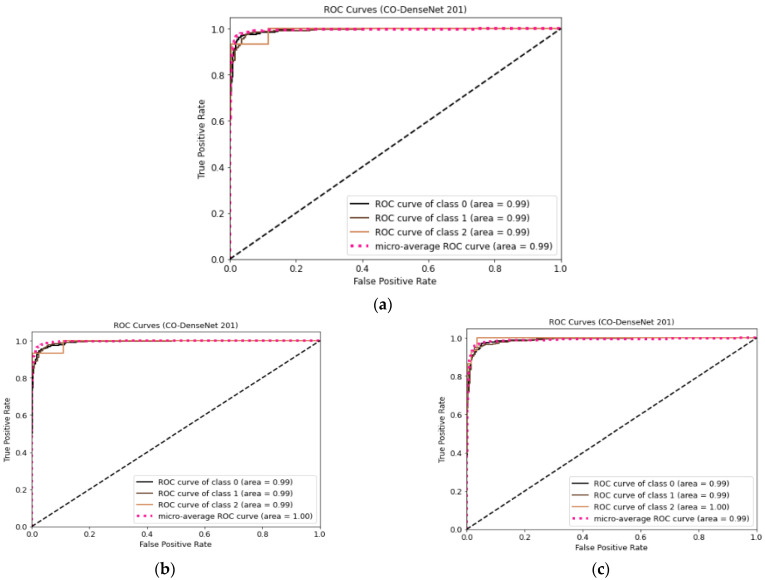
ROC curve for LDDNet: (**a**) Adam optimizer, (**b**) Nadam optimizer, and (**c**) SGD optimizer.

Figure 9 shows the ROC curves for LDDNet for the Adam, Nadam, and SGD optimizers. Here, class “0” indicates normal, “1” indicates pneumonia-affected, and “2” indicates COVID-19-affected X-ray images. From Figure 9, it can be observed that the ROC values for all types of lungs are 99% regardless of the optimizer used, except for COVID-19 cases with an SGD optimizer value of 100%. 

The overall output of all the models for all the applied optimizers is presented in Table 11.

The X-ray dataset described in Table 3 of Section 3 indicates that the number of images for COVID-19 patients is significantly lower than that for pneumonia patients and the normal class. As a result, the X-ray dataset is imbalanced. Effective classification with unbalanced data is a key study subject, as high-class imbalance is present in many real-world applications, such as illness detection. Highly uneven data adds difficulty as it ignores the minority class. The classification accuracy metric is affected by imbalanced data; however, recall is unaffected by imbalanced data. Therefore, the recall value for the results of the X-ray dataset is a useful indicator. 

Next, a balanced X-ray dataset was generated from [58], and the proposed LDDNet was applied to the dataset. This dataset is presented in Table 12. The results for LDDNet for the different optimizers are presented in Table 13. The best classification accuracy for normal, COVID-19, and pneumonia cases is achieved when the Nadam optimizer is applied. 

### 5.3. Comparison of Our Proposed Model with Some Existing Methods

In this section, we compare our proposed modified LDDNet model with some existing models for both the CT scan images and the X-ray images. The proposed LDDNet model supports the reuse of features through dense connections between layers within a dense block. The dense connections improve gradient flow and allow for robust feature propagation between layers. In addition, LDDNet has far fewer trainable parameters, hence enhancing computational efficiency. Adjusting the class weights correspondingly mitigated the bias and skewness produced by the uneven class distribution. In addition, early stopping was utilized to alleviate the overfitting issue while maintaining the generalization capacity of deep neural networks. In addition, the final few layers of LDDNet were fine-tuned to better fit the learned high-level characteristics to the COVID-19 detection task. LDDNet, with these additions, performed exceptionally well in comparison to other models.

Next, the comparison was carried out in terms of the accuracy, recall, precision, AUC, and F1 score and is shown in Table 14. From the table, the accuracy achieved by the proposed model outperforms all other considered models. The work of [5] and [18] also used both X-ray and CT scan images, where [5] achieved 93% and 84.73% accuracy, respectively; however, they performed binary classification. In the work of [20], 98.99% accuracy was achieved using the CO-ResNet model; however, the author only used X-ray images. The author of [35] showed an accuracy of 94.52% for VGG-19, where only one optimizer, RMSprop, was used for CT scan images, while our proposed work used CT scan and X-ray images and the Adam, Nadam, and SGD optimizers. In the work of [36] and [40], a comparatively small dataset was used. The author of [41] used both 2481 CT scan and 1662 X-ray images and achieved 96.18% and 99.40% accuracy, respectively; however, our proposed work used 1043 CT scan images and 5953 X-ray images and achieved 99.36% and 99.55% accuracy, respectively. Since the datasets used in our study and the considered literature are different, a direct comparison was unrealistic. However, our proposed LDDNet model can be considered a potential model for three-class classification of chest CT images and X-ray images with the highest accuracy and precision values.

Table 15 presents the comparative performance of LDDNet with XceptionNet and ResNet152V2 when applied to the balanced X-ray dataset. For the case of the balanced X-ray dataset, the overall accuracy of LDDNet, XceptionNet, and ResNet152V2 is 97.07%, 94.14%, and 95.47%, respectively. This indicates the superiority of LDDNet for the dataset considered.

## 6. Conclusions

This paper introduces a new LDDNet framework to classify infectious diseases in multiclass classification, including pneumonia and COVID-19. For the experiment and to evaluate the model, three types of datasets were used. First, a dataset of 1043 CT scan images was used. Images of COVID-19-affected lungs, pneumonia-affected lungs, and healthy lungs were included in two X-ray datasets: An imbalanced dataset with 5935 images and a balanced dataset with 5002 images. The data was preprocessed by applying data augmentation, rotation, zooming, flipping, and normalization. To enhance the performance of this model, a combination of the global average pooling layer, batch normalization, dense layer, and dropout layer was attached to the base model. Different optimizers, including Adam, Nadam, and SGD, were applied for proper diagnosis and evaluation of each class (COVID-19 vs. normal or non-COVID-19 vs. pneumonia). Among the implemented models, the modified LDDNet showed the best performance for both the CT scan and X-ray datasets, and in each case, Nadam was considered the optimizer. The accuracy of the proposed model was 99.36% for the CT scan images, 99.55% for the imbalanced X-ray dataset, and 97.07% for the balanced X-ray dataset.

With our proposed pretrained deep CNN models, DenseNet, the constraints can be overcome. The models proposed in the current study may detect a COVID-19-positive instance in less than two seconds. With the minimal patient data we had, our proposed models attained an accuracy of over 97 percent. In comparison to recent methods pro-posed by the state-of-the-art technology, it is clear from the discussions that our proposed models obtained encouraging and promising results in detecting COVID-19 in chest X-ray pictures. Data indicates that deep learning will play a significant role soon in combating the COVID-19 outbreak. To validate our model, further patient data must be added to the training dataset. In this study, our proposed chest X-ray image-based models attempted to improve COVID-19 detection. The presented models can greatly minimize physician workload. 

Although our proposed model can detect infectious diseases (pneumonia, COVID-19) from both X-ray and CT scan images, the efficiency of the method depends on the dataset. The prediction of the disease may be incorrect if the dataset contains a significant number of distorted and noisy images. A large dataset is required to train any DL model to increase correct predictions. Therefore, a comparison of our model with others is challenging. However, the proposed LDDNet still has some limitations. It does not make a judgment about the grade for COVID-19. Moreover, it cannot handle the datasets constructed via a mixing of CT and CXR. In our future work, we hope to solve the above problems. In the future, the efficiency of this model should be evaluated for a large dataset and for more than three class classifications in the case of infectious diseases, such as pneumonia, COVID-19, or other viral and bacterial diseases. Furthermore, the experiment can be examined for ultrasound images. Finally, in future work, the enhancement will be conducted for the input image contrast, as this is an important and useful step for improving the visual quality of images. Variations in illumination, random fluctuations in intensity, or inadequate contrast may distort images in many cases. The Gaussian filter will be used in the future to remove image noise and enhance the images within the datasets.

## Figures and Tables

**Table 1:** Summary of the literature review.

Ref.	Modality	Dataset	No. of Classes	Model	Performance Metrics	Limitations
[10]	Lung CT scans	[22]	2	VGG-19	Accuracy: 95.75%, ROC-AUC: 99.30% Recall: 97.13%, F1 score: 95.75,	Lack of automatic hyperparameter optimization techniques, used framework does not allow processing of 3D CT scans.
[11]	CT images	Alexion, Toshiba Medical System, Japan	2	ResNet-101	Accuracy: 99.51%, Sensitivity: 100%, Specificity: 99.02%, AUC: 99.4%	Only considered CT images
[3]	CT images	[23,24]	2	ResNet-18	Accuracy: 99.4%, Sensitivity: 100%, Specificity: 98.6%, AUC: 99.65%	Small dataset and no clinically obtained CT images for COVID-19 infection
[5]	Lung CT scans	[23]	2	Stacked collaboration of VGG-19 and DenseNet-169 models	Accuracy: 84.73% Precision: 79.13% Recall: 92.86% F1 score: 85.45%	Results can be upgraded using preprocessing techniques in different better and efficient ways.
	Lung X-ray images	[22,27,28,29]			Accuracy: 93% Precision: 93% Recall: 93% F1 score: 93%	
[17]	Chest CT scans	Clinical data of various Indian hospitals	2	ANN	Accuracy: 99% AUC: 98.6%	Small raw dataset and not verified for further mutation of COVID-19.
[18]	CT scan Chest X-ray	[24,30,31]	2	CNN-tailored DNN	Accuracy: 96.28% AUC: 98.08% False negative rate: 0.0208	Used CNN-tailored DNN is not computationally efficient.
[19]	CT scan	[24,32]	2	MobileNet	Sensitivity: 96.11% Precision: 96.11% F1 score: 96.11% Accuracy: 94.12%	The experiment can be modified by assembling different pretrained algorithms.
[20]	X-ray image	[33,34]	3	CO-ResNet	Precision: 0.45 Recall: 0.90 F1 score: 0.60 Accuracy: 98.99%	Model is implemented only on X-ray images.
[21]	Lung CT image	[38]	2	NASNet-Mobile	Accuracy: 82.42%, Recall: 78.16%, AUC: 91.00%	Model shows a diverse performance for different datasets.
				NASNet-Large	Accuracy: 81.06%, Recall: 80.06%, AUC: 89.00%	
[35]	CT scan image	[23]	2	VGG-19	Accuracy: 94.52%	Only one optimizer is used for the experiment.
[36]	lung CT	Clinical data collected from a hospital	2	3D DNN	Accuracy: 90.1% PPV: 84% Specificity: 91.10%, AUC: 95.90%, Recall: 90.70%	Absence of temporal information in lung segmentation, data from one hospital only.
[40]	CT scan image	Collected from different hospitals	2	2D CNN	Accuracy: 89.50% Recall: 87%, Specificity: 88%	Relatively small training dataset.
[41]	CT scan images	[32,37]	2	CO-IRv2	Precision: 95.35% Recall: 97.23% F1 score: 96.28% Accuracy: 96.18% AUC: 95%	Relatively small training dataset.
	X-ray images	[42]			Accuracy: 99.40% Recall: 99.38%	
[42]	CT images	Custom dataset collected from 3 hospitals	4	Modified Inception	Specificity: 87% Recall: 88% F1 score: 77% Accuracy: 89.5%	Only CT images were collected from 259 patients. Small training dataset.

**Table 2:** Per class CT scan images applied in the training and testing phases.

Phases	Non-COVID-19	COVID-19	CAP
Training	279	301	306
Testing	54	50	53
Total	333	351	359

**Table 3:** Per class X-ray images applied in training and testing phases.

Phases	Normal	Pneumonia	COVID-19
Training	1338	3642	64
Testing	245	631	15
Total	1583	4273	79

**Table 4:** The dimensions of different layers of LDDNet.

Index	Layers	Parameters of the Layers	Output
1	Input		224 × 224 × 3
2	Zero Padding 2D		230 × 230 × 3
3	Convolution 2D	7 × 7 conv, stride 2	112 × 112 × 64
4	Pooling 2D	3 × 3 max pool, stride 2	56 × 56 × 64
5	Dense Block 1	$\left[\begin{array}{c} 1\times 1\;\mathrm{conv}\\ 3\times 3\;\mathrm{conv} \end{array}\right]$× 6	56 × 56 × 256
6	Transition Layer 1	1 × 1 conv	56 × 56 × 256
		2 × 2 average pool, stride 2	28 × 28 × 128
7	Dense Block 2	$\left[\begin{array}{c} 1\times 1\;\mathrm{conv}\\ 3\times 3\;\mathrm{conv} \end{array}\right]$× 12	28 × 28 × 512
8	Transition Layer 2	1 × 1 conv	28 × 28 × 512
		2 × 2 average pool, stride 2	14 × 14 × 256
9	Dense Block 3	$\left[\begin{array}{c} 1\times 1\;\mathrm{conv}\\ 3\times 3\;\mathrm{conv} \end{array}\right]$× 48	14 × 14 × 1792
10	Transition Layer 3	1 × 1 conv	14 × 14 × 1792
		2 × 2 average pool, stride 2	7 × 7 × 896
11	Dense Block 4	$\left[\begin{array}{c} 1\times 1\;\mathrm{conv}\\ 3\times 3\;\mathrm{conv} \end{array}\right]$× 32	7 × 7 × 1920
12	Global Average Pooling	7 × 7 global average pool	1 × 1 × 1920
12	Batch Normalization		1 × 1 × 1920
13	Dense Layer		1 × 1 × 1024
14	Dropout Layer		1 × 1 × 1024
15	Dense Layer		1 × 1 × 512
16	Dense Layer		1 × 1 × 256
17	Batch Normalization		1 × 1 × 256
18	Dropout		1 × 1 × 256
19	Dense Layer		1 × 1 × 3

**Table 5:** Comparison of different DL models for the Adam optimizer.

Model	Class	PPV	Recall	F1 Score	Accuracy
ResNet152V2	Non-COVID-19	98%	100%	99%	99.36%
	COVID-19	100%	98%	99%	99.36%
	CAP	100%	100%	100%	100%
LDDNet	Non-COVID-19	98%	100%	99%	99.36%
	COVID-19	100%	98%	99%	99.36%
	CAP	100%	100%	100%	100%
XceptionNet	Non-COVID-19	94%	94%	94%	96.18%
	COVID-19	94%	94%	94%	96.18%
	CAP	100%	100%	100%	100%

**Table 6:** Comparison of the different DL models for the Nadam optimizer.

Model	Class	PPV	Recall	F1 Score	Accuracy
ResNet152V2	Non-COVID-19	96%	100%	98%	98.73%
	COVID-19	98%	94%	96%	97.45%
	CAP	98%	98%	98%	98.73%
LDDNet	Non-COVID-19	100%	100%	100%	100%
	COVID-19	100%	98%	99%	99.36%
	CAP	98%	100%	99%	99.36%
XceptionNet	Non-COVID-19	96%	94%	95%	96.82%
	COVID-19	94%	96%	95%	96.82%
	CAP	100%	100%	100%	100%

**Table 7:** Comparison of different DL models for the SGD optimizer.

Model	Class	PPV	Recall	F1 Score	Accuracy
ResNet152V2	Non-COVID-19	96%	94%	95%	96.82
	COVID-19	90%	94%	92%	94.9%
	CAP	98%	96%	97%	98.09%
LDDNet	Non-COVID-19	98%	98%	98%	98.73%
	COVID-19	98%	98%	98%	98.73%
	CAP	100%	100%	100%	100%
XceptionNet	Non-COVID-19	89%	93%	91%	93.63%
	COVID-19	94%	88%	91%	94.27%
	CAP	98%	100%	99%	99.36%

**Table 8:** Comparison of the different DL models for the Adam optimizer.

Model	Class	PPV	Recall	F1 Score	Accuracy
ResNet152V2	Normal	92%	93%	92%	95.74%
	Pneumonia	97%	97%	97%	96.18%
	COVID-19	92%	80%	86%	99.55%
LDDNet	Normal	93%	96%	95%	96.86%
	Pneumonia	98%	97%	98%	96.75%
	COVID-19	90%	60%	72%	99.21%
XceptionNet	Normal	88%	93%	90%	94.61%
	Pneumonia	97%	95%	96%	94.61%
	COVID-19	100%	87%	93%	99.78%

**Table 9:** Comparison of different DL models for the Nadam optimizer.

Model	Class	PPV	Recall	F1 Score	Accuracy
ResNet152V2	Normal	91%	93%	92%	95.62%
	Pneumonia	97%	96%	97%	95.51%
	COVID-19	72%	87%	79%	99.21%
LDDNet	Normal	94%	95%	94%	96.75%
	Pneumonia	98%	98%	98%	96.75%
	COVID-19	100%	73%	85%	99.55%
XceptionNet	Normal	87%	93%	90%	94.5%
	Pneumonia	97%	95%	96%	94.61%
	COVID-19	100%	80%	89%	99.66%

**Table 10:** Comparison of the different DL models for the SGD optimizer.

Model	Class	PPV	Recall	F1 Score	Accuracy
ResNet152V2	Normal	83%	94%	88%	93.04%
	Pneumonia	96%	93%	94%	92.26%
	COVID-19	90%	70%	80%	98.32%
LDDNet	Normal	87%	97%	92%	95.29%
	Pneumonia	99%	95%	97%	95.4%
	COVID-19	92%	73%	81%	99.44%
XceptionNet	Normal	79%	89%	83%	90.35%
	Pneumonia	93%	91%	92%	88.89%
	COVID-19	90%	70%	80%	98.32%

**Table 11:** Overall performance of the experimental models applied to different datasets.

Dataset	Model	Optimizers		
		Adam	Nadam	SGD
CT Scan Images	ResNet152V2	99.36%	97.45%	94.90%
	LDDNet	99.36%	99.36%	98.73%
	XceptionNet	96.18%	96.82%	93.63%
X-ray Images	ResNet152V2	95.74%	95.17%	91.81%
	LDDNet	96.41%	99.55%	95.06%
	XceptionNet	94.5%	94.36%	88.78%

**Table 12:** Image distribution in the balanced X-ray dataset.

Class	Training	Testing
Normal	1541	261
COVID-19	1527	217
Pneumonia	1183	273
Total	4251	751

**Table 13:** Comparison of different optimizers for LDDNet for the case of the balanced X-ray dataset.

Model	Class	PPV	Recall	F1 Score	Accuracy
Adam	Normal	99%	90%	94%	95.87%
	COVID-19	98%	100%	99%	99.47%
	Pneumonia	90%	98%	94%	95.87%
Nadam	Normal	96%	96%	96%	97.20%
	COVID-19	100%	100%	100%	99.87%
	Pneumonia	96%	96%	96%	97.07%
SGD	Normal	98%	91%	94%	95.87%
	COVID-19	97%	100%	99%	99.20%
	Pneumonia	93%	97%	95%	96.40%

**Table 14:** Comparison of our proposed model with some existing literature.

Ref.	Methods	Type of Image	Accuracy	Recall	Precision	F1 Score	AUC
[5]	Ensemble of VGG-19 and DenseNet169	CT scan, X-ray	84.73%, 93%	92.86%	79.13%	85.45%	-
[10]	VGG-19	CT scan	95.75%	97.13%	-	95.75%	99.30%
[18]	CNN-tailored DNN	CT scan, X-ray	96.28%	-	-	-	98.08%
[19]	MobileNet	CT scan	94.12%	96.11%	96.11%	96.11%	-
[20]	CO-ResNet	X-ray	98.99%	90%	45%	60%	-
[21]	NASNet-Mobile	CT scan	82.42%	78.16%	-	-	91.00%
[35]	VGG-19	CT scan	94.52%	-	-	-	-
[36]	U-Net CNN	CT scan	-	90.70%	-	-	95.90%
[40]	2D CNN	CT scan	89.50%	87%	-	-	-
[41]	CO-IRv2	CT scan	96.18%	97.23%	95.35%	96.28%	95%
		X-ray	99.40%	99.38%	100%	99.69%	
[43]	Modified XceptionNet	CXR	99.53%	77%	100%	87%	-
Proposed Model	LDDNet	CT scan	99.36%	98%	100%	99%	99.98%
	LDDNet	X-ray	99.55%	73%	100%	85%	99.56%

**Table 15:** Comparison of different DL models for the Nadam optimizer for X-ray dataset.

Model	Class	PPV	Recall	F1 Score	Accuracy
ResNet152V2	Normal	95%	92%	94%	95.61%
	COVID-19	100%	99%	99%	99.6%
	Pneumonia	92%	96%	94%	95.74%
LDDNet	Normal	96%	96%	96%	97.20%
	COVID-19	100%	100%	100%	99.87%
	Pneumonia	96%	96%	96%	97.07%
XceptionNet	Normal	97%	89%	93%	94.94%
	COVID-19	98%	99%	98%	98.93%
	Pneumonia	88%	96%	92%	94.41%

## Data Availability

The data used to support the findings of this study are available from the corresponding author (M.R.H.M.) upon request.
